# A Possible Role of *cis*‐8‐Octadecenoic Acid of the Sebum in Facial Skin Redness

**DOI:** 10.1111/jocd.16570

**Published:** 2024-09-15

**Authors:** Arisa Kato, Eri Shimizu, Hisashi Tsujimura, Akane Kawamoto, Etsuko Watarai, Takanori Igarashi, Hiroyuki Yoshida

**Affiliations:** ^1^ Biological Science Research Kao Corporation Kanagawa Japan; ^2^ Analytical Science Research Kao Corporation Tochigi Japan; ^3^ Skin Care Products Research Kao Corporation Kanagawa Japan

**Keywords:** *cis*‐8‐octadecenoic acid, erythema, interleukin‐36γ, interleukin‐37, oleic acid, skin redness, skin sebum

**Abbreviations**
EI: erythema index
FFA: free fatty acid
NMDA: *N*‐methyl‐D‐aspartate

Dear Editor,

Sebum on the skin surface is essential for maintaining the homeostasis and function of human skin; however, excessive sebum secretion and disturbances in sebum composition are known to cause various chronic inflammatory skin disorders [1]. Free fatty acids (FFAs), significant components of human sebum, consist mostly of saturated and monoenoic FFAs with chain lengths of 16 or 18 carbons [2]. Recently, we showed that the proportion of FFAs (C18:1) in sebum positively correlated with cheek redness in healthy individuals. Furthermore, oleic acid (C18:1, *cis*‐9), one of the best‐characterized sebum FFAs that induce interleukin‐36γ expression, may be a link between skin redness and sebum [3]. In contrast, *cis*‐8‐octadecenoic acid (C18:1, *cis*‐8) was first identified in 1974 as the most abundant isomer of sebum FFAs (C18:1) [4, 5]; however, its physiological functions remain elusive, likely owing to its lower commercial availability compared with other FFAs. Here, we investigated the relationship between *cis*‐8‐octadecenoic acid and cheek redness in healthy Japanese individuals, and its effects on interleukin‐36γ expression in cultured human normal keratinocytes (see the online Supporting materials and methods).

We first confirmed that *cis*‐8‐octadecenoic acid was the most abundant species among the four FFA (C18:1) isomers in skin sebum (Figure 1A). Next, we showed that the percentage composition of *cis*‐8‐octadecenoic acid in sebum was positively correlated with skin biophysical parameters associated with facial skin redness, that is, erythema index (EI) and a* values (Figure 1B,C), suggesting that the proportion of *cis*‐8‐octadecenoic acid in sebum is related to skin redness. Figure 1D shows representative images of the cheek of participants who had high or low degrees of skin redness and *cis*‐8‐octadecenoic acid proportion in the sebum. Recently, the ratio of the pro‐inflammatory cytokine interleukin‐36γ to the anti‐inflammatory cytokine interleukin‐37 in the stratum corneum was reported as a new skin inflammatory index that positively correlates with the degree of facial skin redness [6]. Therefore, to investigate the mechanism underlying the involvement of *cis*‐8‐octadecenoic acid in skin redness, we evaluated the dose‐ and time‐dependent effects of *cis*‐8‐octadecenoic acid on the *interleukin‐36*γ/*interleukin‐37* ratio in human epidermal keratinocytes. Addition of *cis*‐8‐octadecenoic acid to culture medium dose‐dependently induced *interleukin‐36*γ and *interleukin‐37* mRNA expression and the *interleukin‐36*γ*/interleukin‐37* ratio, showing the highest elevation at dose of 150 μM compared with that of vehicle control (Figure 2A–C). Notably, oleic acid significantly increased *interleukin‐36*γ mRNA expression and the *interleukin‐36*γ/*interleukin‐37* ratio at dose of 50 μM, whereas *cis*‐8‐octadecenoic acid at this dose showed no significant induction (Figure 2A,C). These results indicated that *cis*‐8‐octadecenoic acid has a more moderate inflammatory effect than oleic acid. Time‐course analyses showed that the *cis*‐8‐octadecenoic acid (100 or 150 μM)‐induced enhancement of the *interleukin‐36*γ*/interleukin‐37* ratio started after 3 h and reached maximal levels 24 h after treatment (Figure 2D). Previously, we demonstrated that *N*‐methyl‐D‐aspartate (NMDA)‐type glutamate receptors are involved in the oleic acid‐induced *interleukin*‐*36*γ*/interleukin‐37* ratio in human epidermal keratinocytes [3]. To investigate the involvement of NMDA‐type glutamate receptors in the *cis*‐8‐octadecenoic acid‐induced *interleukin‐36*γ/*interleukin‐37* ratio, we examined the effects of MK801, a potent NMDA receptor antagonist, on *interleukin‐36*γ and *interleukin‐37* mRNA expression in *cis*‐8‐octadecenoic acid‐treated keratinocytes. As shown in Figure 2E, *cis*‐8‐octadecenoic acid‐induced *interleukin‐36*γ/*interleukin‐37* ratio was suppressed by MK801 in a dose‐dependent manner. These results suggest that, similar to oleic acid, *cis*‐8‐octadecenoic acid upregulates the *interleukin‐36*γ/*interleukin‐37* ratio in part through NMDA‐type glutamate receptor, which may lead to increased skin redness. Although further studies are required, the variations in the binding affinity of *cis*‐8‐octadecenoic acid and oleic acid for the receptor may be attributed to their distinct inflammatory effects.

**FIGURE 1:**
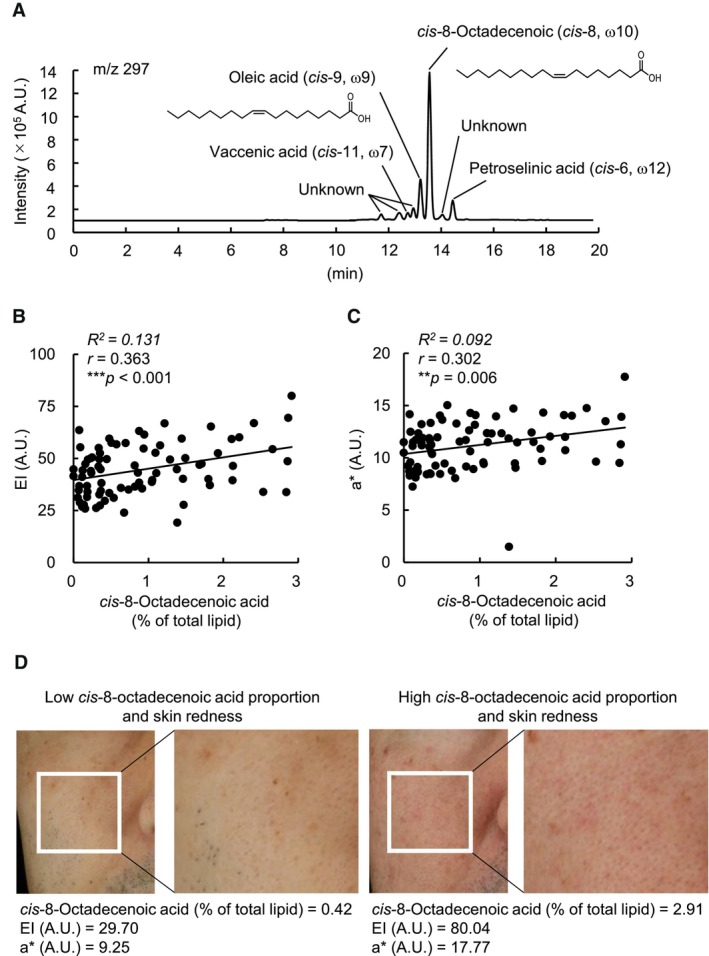
Correlation between *cis*‐8‐octadecenoic acid composition in sebum and cheek redness in healthy individuals. (A) Representative chromatogram of *m*‐chloroperoxybenzoic acid (mCPBA)‐epoxidized monounsaturated fatty acid isomers comprising vaccenic acid (C18:1, *cis*‐11), oleic acid (C18:1, *cis*‐9), *cis*‐8‐octadecenoic acid (C18:1, *cis*‐8), and petroselinic acid (C18:1, *cis*‐6) in skin sebum. *Insets*, structures of *cis*‐8‐octadecenoic acid and oleic acid. (B and C) Scatter plot of *cis*‐8‐octadecenoic acid percentage composition versus the EI (B) or a* values (C) is shown. Values for *cis*‐8‐octadecenoic acid were calculated as percentages in the total lipid content. Pearson's correlation coefficient analysis was used to examine the relationship between them. *R* ^2^, coefficient of determination, *r*, correlation coefficient; ***, *p <* 0.001; **, *p <* 0.01. (D) Representative images of participant's facial skin showing low degrees of skin redness and *cis*‐8‐octadecenoic acid proportion in the sebum (*left*) and high degrees of skin redness and *cis*‐8‐octadecenoic acid proportion in the sebum (*right*).

**FIGURE 2:**
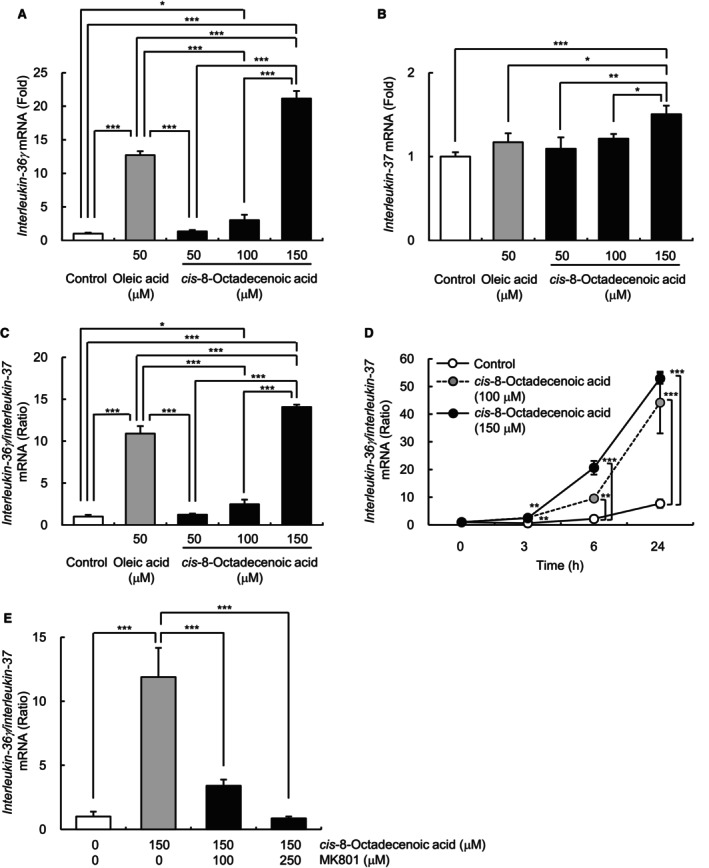
Effects of *cis*‐8‐octadecenoic acid and MK801 on *interleukin‐36*γ and *interleukin‐37* mRNA expression and *interleukin‐36*γ/*interleukin‐37* ratio in cultured normal human keratinocytes. (A–C) Dose‐dependent expression of *interleukin‐36*γ (A) and *interleukin‐37* (B) mRNA and the *interleukin‐36*γ*/interleukin‐37* ratio (C). *cis*‐8‐octadecenoic acid (0, 50, 100, or 150 μM) or oleic acid (50 μM) were applied to keratinocytes, and mRNA levels were measured 8 h later. The values are expressed as means ± standard deviation (SD, *n =* 3) and presented as fold elevation in mRNA expression relative to that in control cells. *Control*, cells treated with vehicle (ethanol) alone. Tukey's multiple comparison test was used for statistical analysis. ***, *p <* 0.001; **, *p <* 0.01; *, *p <* 0.05. (D) Time‐dependent expression of the *interleukin‐36*γ*/interleukin‐37* ratio. *cis*‐8‐octadecenoic acid (0, 100, or 150 μM) was applied to keratinocytes, and mRNA levels were measured 3, 6, or 24 h later. The values are expressed as means ± SD (*n =* 3) and presented as fold elevation in mRNA expression relative to that in control cells. Dunnett's test was used for statistical analysis. ***, *p <* 0.001; **, *p <* 0.01. (E) Effects of MK801 on the *cis*‐8‐octadecenoic acid‐induced *interleukin‐36*γ*/interleukin‐37* ratio. Keratinocytes were treated with or without *cis*‐8‐octadecenoic acid (150 μM) for 7 h in the absence or presence of MK801 (100 or 250 μM). The values are expressed as means ± SD (*n =* 3) and presented as fold elevation in mRNA expression relative to that in control cells in the absence of MK801. *Control*, cells treated with vehicle (50% ethanol in phosphate‐buffered saline) alone. Tukey's multiple comparison test was used for statistical analyses. ***, *p <* 0.001.

In conclusion, to the best of our knowledge, our study is the first to show that *cis*‐8‐octadecenoic acid plays a role in facial skin redness.

## Author Contributions

A. Kato, E. Watarai, T. Igarashi, and H. Yoshida designed the research. A. Kato, A. Kawamoto, E. Watarai, E. Shimizu, and T. Igarashi performed the research. A. Kato, A. Kawamoto, E. Shimizu, H. Tsujimura, E. Watarai, T. Igarashi, and H. Yoshida analyzed the data. A. Kato, E. Shimizu, and H. Yoshida wrote the manuscript. All the authors approved the final manuscript.

## Ethics Statement

The study was conducted under the Declaration of Helsinki and was approved by the Ethical Committee of Kao Corporation (approval no. D123‐200512).

## Conflicts of Interest

The authors declare no conflicts of interest.

## Supporting information


Data S1.

## Data Availability

The authors have nothing to report.
