# Supplementary material for: A Possible Role of cis‐8‐Octadecenoic Acid of the Sebum in Facial Skin Redness
Source: J Cosmet Dermatol. 2024 Sep 15;24(1):e16570. doi: 10.1111/jocd.16570 (PMC11743226; doi:10.1111/jocd.16570)
Supplement: Supplementary file 1 — Data S1. [file JOCD-24-e16570-s001.docx]

**Supporting materials and methods**

***Reagents***

Oleic acid was purchased from Sigma-Aldrich (St. Louis, MO, USA) and *cis*-8-octadecenoic acid and vaccenic acid were purchased from Cayman Chemical Company (Ann Arbor, MI, USA). Petroselinic acid was purchased from Toronto Research Chemicals (Toronto, Canada), lauric acid (Methyl-d3) was purchased from Cambridge Isotope Laboratories (Tewksbury, MA, USA), 3-chloroperbenzoic acid was purchased from Sigma-Aldrich, and MK801 (dizocilpine; (5S,10R)-(+)-5-methyl-10,11-dihydro-5H-dibenzo [a,d] cyclohepten-5,10-imine) was purchased from Fujifilm Wako Pure Chemical Industries (Osaka, Japan).

***Human clinical study***

For this study, 81 healthy Japanese individuals (age range, 20–55 years; mean age ± S.D., 39.98 ± 10.24 years; 52 females and 29 males) were recruited. Each participant was acclimated in a room at 24 °C with constant humidity (40%) for at least 10 min prior to analysis. The study was conducted in accordance with the Declaration of Helsinki and approved by the Ethical Committee of Kao Corporation (approval number D123-200512). Participants provided written informed consent and received an adequate explanation of the processes and motivations of the study. Individuals were excluded if they had any skin diseases, allergies, facial eczema, or wounds that could influence skin redness.

***Measurements of skin redness parameters***

The erythema index (EI) and a* value (for the D65 illuminant) at the cheeks were obtained from spectral reflectance using a CM-2600d spectrophotometer system (Konica-Minolta Inc., Tokyo, Japan), and the EI was calculated using a previously reported formula^1^. Facial images of each participant were obtained using the VISIA-CR skin analysis imaging system (Canfield Scientific, Fairfield, NJ, USA).

***Sebum analysis***

After 90 min of face washing, sebum was collected from the cheeks using cigarette paper (RIZLA: RIZLA BLUE DOUBLE, 1.7 × 1.7 cm). The paper was immersed in a screw tube containing ethanol (Kanto Chemical Co., Inc., Tokyo, Japan; high-performance liquid chromatography [HPLC] grade > 99.8%), and the solvent was evaporated under a N_2_ flow. Sebum was extracted with 1 mL of a mixture of methanol (Kanto Chemical Co., Inc., HPLC grade >99.8%) and chloroform (Kanto Chemical Co., Inc., HPLC grade > 99.7%) (vol/vol = 1/1) for 5 min using sonication. An internal standard solution containing 1 μM trilaurin (triacylglycerol, C36:0; Nu-Chek Prep, Inc., Elysian, MN, USA), 10 μM ditridecanoin (diacylglycerol, C26:0; Larodan Fine Chemicals AB), 100 μM lauric acid (methyl-d3) (free fatty acid [FFA], C12:0-d3; Cambridge Isotope Laboratories), 10 μM lauryl palmitoleate (wax ester, C28:1; Santa Cruz Biotechnology, Inc., Dallas, TX, USA), 10 μM cholesteryl acetate (cholesterol ester, C2:0; Olbracht Serdary Research Laboratories, Toronto, Canada), and 5 μM cholesteryl caprate (cholesterol ester, C10:0; Nu-Chek Prep, Inc., Elysian, MN, USA) was prepared in chloroform. An aliquot (10 μL) of the internal standard solution was dried through evaporation and the residue was dissolved in 50 μL of the sebum solution. Mass spectrometric analysis of sebum was conducted using flow injection coupled with mass spectrometry analysis using a Vanquish Ultra-High-Performance Liquid Chromatography (UHPLC) system (Thermo Fisher Scientific, Waltham, MA, USA) coupled with a Q-Exactive Focus system (Thermo Fisher Scientific) equipped with a heated electrospray source. Chloroform/methanol (vol/vol = 1/1) containing 15 mM ammonium acetate (Fujifilm Wako Pure Chemicals, Guaranteed Reagent) was used as the mobile phase at a flow rate of 0.1 mL/min. The optimized experimental design parameters were set as follows: voltages in the positive and negative ion modes, 3.5 and 2.5 kV, respectively; auxiliary gas, 10 psi; sheath gas pressure, 35 psi; heated capillary temperature, 250 °C; and heated vaporizer temperature, 200 °C. Fourier transform mass spectrometry (FT-MS) was performed in full-scan mode, and the set parameters were as follows: resolution, 70,000; auto-gain control target, <1 × 10^6^; and m/z range, 180–1200. The system was controlled using Xcalibur 4.1.31.9 software (Thermo Fisher Scientific). Raw data were analyzed using TraceFinder 4.1 software (Thermo Fisher Scientific). The total amount of lipids in the sebum was calculated as the sum of the quantities of each lipid constituent.

***Monounsaturated fatty acid (MUFA) isomer analysis***

The sebum solution was concentrated 5-fold and used for MUFA isomer analysis. Epoxidation derivatization of the lipid extracts for MUFA isomer analysis was performed based on a previously reported method^2^. Briefly, 50 μL of lipid extract was mixed with 50 μL of epoxidation reagent (200 mM *m*-chloroperoxybenzoic acid [mCPBA; Sigma-Aldrich] dissolved in acetonitrile/isopropanol/water, vol/vol/vol: 65/30/5) and derivatized. The mixture was then incubated at 55 °C for 1 h to ensure complete reaction. An internal standard solution containing 25 mM lauric acid (methyl-d3, FFA, C12:0-d3; Cambridge Isotope Laboratories) was prepared in chloroform. An aliquot of 10 μL of the internal standard solution was evaporated to dryness and dissolved in 50 μL of the derivatized sebum sample supernatant. The FFAs and epoxy-MUFAs were analyzed using an Agilent 6130 Series Liquid Chromatography/Mass Spectrometry (LC/MS) SL system equipped with an electrospray ionization (ESI)-ion source, ChemStation software, and an Agilent 1260 Infinity Series LC system (Agilent Technologies, Santa Clara, CA, USA). Chromatographic separation of the lipids was performed using an Acquity UPLC BEH C18 system (2.1 × 100 mm; particle size, 1.7 μm; Waters Corporation, Milford, MA, USA) at a flow rate of 0.2 mL/min using a binary gradient solvent system of mobile phase A (ultrapure water/methanol [vol/vol = 70/30] containing 0.5 mM ammonium acetate and 0.5 mM ammonium hydrogen carbonate) and mobile phase B (ultrapure water/tetrahydrofuran/acetonitrile [vol/vol/vol = 5/10/85] containing 0.5 mM ammonium acetate and 0.5 mM ammonium hydrogen carbonate). The mobile phases were programmed as follows: 0–30% B (0–2 min), 30–93% B (2–32 min), 93% B (32–42 min), and 0% B (42–55 min). The sample injection volume was 5 μL and the column temperature was maintained at 40 ℃. The mass spectrometric parameters were set as follows: polarity, negative ion mode; flow of heated dry N_2_ gas, 4.0 L/min; nebulizer gas pressure, 50 psi; heater temperature of N_2_ gas, 350 ℃; vaporizer temperature, 350 ℃; capillary voltage, 3500 V; and fragmentor voltage, 150 V for FFA and 100 V for epoxy-MUFA. FFAs and epoxy-MUFAs were detected using selected ion monitoring at m/z [M-H]–. Raw data were analyzed using an Agilent Open LAB CDS ChemStation C.01.07 SR1 system (Agilent Technologies).

***Cell culture***

Normal human epidermal keratinocytes (Kurabo, Osaka, Japan) were routinely cultured in HuMedia-KG2 medium containing human keratinocyte growth supplement (Kurabo) in a humidified atmosphere with 5% CO_2_ at 37 °C. Upon reaching confluence, the cells were cultured in HuMedia-KG2 medium containing human keratinocyte growth supplement without human epidermal growth factor (Kurabo)　and bovine pituitary extract (Kurabo), and treated with lipids and MK801 dissolved in 50% ethanol in phosphate-buffered saline (Fujifilm Wako Pure Chemicals).

***Real-time quantitative polymerase chain reaction (qPCR)***

Total RNA was isolated from keratinocytes using the RNeasy Mini Kit (Qiagen, Hilden, Germany) with DNase treatment and a QIAcube Connect system (Qiagen, Hilden, Germany). cDNA was synthesized using a QuantiTect Reverse Transcription Kit (Qiagen, Hilden, Germany). Real-time qPCR was performed using the TaqMan Fast Advanced Master Mix (Thermo Fisher Scientific). TaqMan probes were purchased from Applied Biosystems (Foster City, CA, USA), and the relative quantification values of *interleukin-36*γ (Hs00219742_m1) and *interleukin-37* (Hs00367201_m1) were normalized against ribosomal protein lateral stalk subunit P0 (*RPLP0*) (Hs99999902_m1) using the QuantStudio 5 Real-Time PCR Systems (Applied Biosystems).

***Statistical analysis***

Data were compared using a one-way analysis of variance (ANOVA), followed by Tukey’s test or Dunnett’s test for multiple comparisons. Correlations were assessed using Pearson’s correlation coefficients. Statistical significance was set at *p* < 0.05. All statistical analyses were performed using IBM SPSS Statistics 28.0.0.0 (IBM, NY, USA) or Microsoft Excel (Office 365) (Microsoft, WA, USA) software.

**References**

1. Dawson JB, Barker DJ, Ellis DJ, Grassam E, Cotterill JA, Fisher GW, Feather JW, A theoretical and experimental study of light absorption and scattering by in vivo skin. *Phys Med Biol.* 1980; 25: 695-709.

2. Kuo TH, Chung HH, Chang HY, Lin CW, Wang MY, Shen TL, Hsu CC, Deep lipidomics and molecular imaging of unsaturated lipid isomers: A universal strategy initiated by mCPBA epoxidation. *Anal Chem.* 2019; 91: 11905-11915.
